# Draft Genomes, Phylogenetic Reconstruction, and Comparative Genomics of Two Novel Cohabiting Bacterial Symbionts Isolated from *Frankliniella occidentalis*

**DOI:** 10.1093/gbe/evv136

**Published:** 2015-07-21

**Authors:** Paul D. Facey, Guillaume Méric, Matthew D. Hitchings, Justin A. Pachebat, Matt J. Hegarty, Xiaorui Chen, Laura V.A. Morgan, James E. Hoeppner, Miranda M.A. Whitten, William D.J. Kirk, Paul J. Dyson, Sam K. Sheppard, Ricardo Del Sol

**Affiliations:** ^1^Institute of Life Sciences, College of Medicine, Swansea University, United Kingdom; ^2^Institute of Biological, Environmental & Rural Sciences (IBERS), Aberystwyth University, Penglais, Ceredigion, United Kingdom; ^3^School of Life Sciences, Keele University, Staffordshire, United Kingdom; ^4^MRC CLIMB Consortium, Institute of Life Science, Swansea University, United Kingdom; ^5^Department of Zoology, University of Oxford, United Kingdom

**Keywords:** thrips, BFo, *Frankliniella*, symbiont, genome, evolution

## Abstract

Obligate bacterial symbionts are widespread in many invertebrates, where they are often confined to specialized host cells and are transmitted directly from mother to progeny. Increasing numbers of these bacteria are being characterized but questions remain about their population structure and evolution. Here we take a comparative genomics approach to investigate two prominent bacterial symbionts (BFo1 and BFo2) isolated from geographically separated populations of western flower thrips, *Frankliniella occidentalis.* Our multifaceted approach to classifying these symbionts includes concatenated multilocus sequence analysis (MLSA) phylogenies, ribosomal multilocus sequence typing (rMLST), construction of whole-genome phylogenies, and in-depth genomic comparisons. We showed that the BFo1 genome clusters more closely to species in the genus *Erwinia,* and is a putative close relative to *Erwinia aphidicola*. BFo1 is also likely to have shared a common ancestor with *Erwinia pyrifoliae/Erwinia amylovora* and the nonpathogenic *Erwinia tasmaniensis* and genetic traits similar to *Erwinia billingiae*. The BFo1 genome contained virulence factors found in the genus *Erwinia* but represented a divergent lineage. In contrast, we showed that BFo2 belongs within the Enterobacteriales but does not group closely with any currently known bacterial species. Concatenated MLSA phylogenies indicate that it may have shared a common ancestor to the *Erwinia* and *Pantoea* genera, and based on the clustering of rMLST genes, it was most closely related to *Pantoea ananatis* but represented a divergent lineage. We reconstructed a core genome of a putative common ancestor of *Erwinia* and *Pantoea* and compared this with the genomes of BFo bacteria. BFo2 possessed none of the virulence determinants that were omnipresent in the *Erwinia* and *Pantoea* genera. Taken together, these data are consistent with BFo2 representing a highly novel species that maybe related to known *Pantoea*.

## Introduction

The *Erwinia* and *Pantoea* genera, within the Enterobacteriaceae, contain common human pathogens, insect symbionts, and phytopathogens (Baumler et al. 2013). There is increasing evidence that previously unknown bacteria isolated from a wide variety of insects belong to this family of bacteria (Husník et al. 2011). These include bacterial symbionts of insects found across a broad range of niches (Allen et al. 2007). However, due to the vastness of the many insect orders and the often highly rearranged genomes of even closely related bacterial species, a comprehensive understanding of these relationships remains to be defined.

Nevertheless, there are a limited number of studies reporting genetic information on the symbiotic relationships between bacteria and their arthropod hosts—helped by technical advances in whole-genome sequencing and analysis. Thus, researchers are now able to amass a significant volume of information on the evolution and relatedness of insect symbiotic lineages. For example, comparative genomics of heritable insect endosymbionts often reveals large scale reductive evolution and fast evolving genomes (Van Ham et al. 2003), often associated with endosymbioses. Despite the emerging picture that other symbiotic bacteria may also be undergoing similar evolutionary processes (Nikoh et al. 2011), many questions remain, particularly in reporting genomic features of gut residing bacteria (Kikuchi et al. 2009).

The western flower thrips (WFT), *Frankliniella occidentalis* (Pergande) (Thysanoptera: Thripidae), is a globally distributed insect pest causing significant damage to greenhouse-grown crops. Thrips infestations typically lead to reduced aesthetics and lowered yield (Kirk and Terry 2003), principally because their method of feeding causes damage to leaves and fruit, thus reducing the marketability of commercial crops. Moreover, WFT carry tospoviruses, including tomato spotted wilt virus (TSWV) (Jensen 2000a, 2000b; Pappu et al. 2009) and, although WFT are asymptomatic carriers of TSWV, it has been estimated that the annual loss to agriculture caused by TSWV alone amounts to $1 billion (Prins and Goldbach 1998). In addition, WFT also harbor two bacterial symbionts that have been shown to reside within the gut lumen. de Vries, Jacobs, et al. (2001) explored the hindgut of each life-stage of WFT and reported two predominant bacterial symbionts—designated BFo1 and BFo2 (Bacteria, *F. occidentalis*). Interestingly, transmission of these bacteria occurs during oviposition and probing during feeding; especially where the sites of feeding and egg laying are infected with BFo-contaminated thrips feces (de Vries, Jacobs, et al. 2001; de Vries 2010). However, for the latter, it is unclear how long, if at all, BFo bacteria are able to survive outside the host. Nevertheless, BFo bacteria appear to be important to *F. occidentalis*. Indeed, the fact that BFo bacteria have been isolated from geographically isolated, wild and greenhouse populations of *F. occidentalis*—including California, Germany, the Netherlands and the United Kingdom (Chanbusarakum and Ullman 2008, 2009; and this study)—provides strong evidence for this being a symbiotic relationship rather than simply a transient occurrence. However, the fact that these bacteria are culturable under laboratory conditions also suggests that they might not be entirely host dependent.

Molecular characterization of the BFo symbiotic bacteria using 16S rRNA and biochemical analysis using API 20E Biochemical tests (Chanbusarakum and Ullman 2008) showed that they had only 95% rRNA sequence identity and shared only 50% of the biochemical properties tested for including catalase, glucose, and mannitol fermentation (Chanbusarakum and Ullman 2008). This suggests divergent evolutionary histories and may indicate that they belong to separate genera. However, the exact classification of these two species has yet to be adequately addressed. For example, there is conflict as to their phylogeny, with de Vries, Breeuwer, et al. (2001) suggesting that BFo1 and BFo2 constitute a monophyletic group with *Escherichia coli*; yet Chanbusarakum and Ullman (2008) suggest that BFo1 groups within the genus *Erwinia*, whereas BFo2 lies separately. Thus, the exact classification of these species is a source of controversy. However, both previous studies on the classification of these symbionts have relied solely on single-gene, 16S rRNA phylogenies which often suffer from saturation in deeply diverged families.

We offer a phylogenomic analysis of the two prominent bacterial symbionts (BFo1 and BFo2) of *F. occidentalis.* Our study focuses first on the classification of these isolates using an in-depth phylogenetic approach and second on their genome evolution and relatedness to existing bacterial species. We reconstruct a core genome for the common ancestor to the *Erwinia*–*Pantoea* clade and compare this with both BFo genomes. Taken together, our analysis allows us to reconstruct a possible evolutionary history of the two prominent bacterial symbionts of *F. occidentalis.*

## Materials and Methods

### Isolation and Culturing of *F. occidentalis* Symbiotic Bacteria

Symbiotic bacteria, previously designated BFo1 and BFo2 (Chanbusarakum and Ullman 2008), were isolated from the following two populations of *F. occidentalis:* 1) A greenhouse population from the Netherlands and 2) a population that has been isolated and maintained at Keele University (Newcastle-under-Lyme, UK). This latter population was established in 1996 after collection of *F. occidentalis* from a UK commercial *Chrysanthemum* nursery in southern England (UK) and maintained since on flowering *Chrysanthemum* plants. Approximately 20 surface sterilized insects were homogenized in 1× TE using a micropestle. Sterilization was performed by the method outlined in de Vries, Breeuwer, et al. (2001). Serial dilutions of the homogenate were plated on LB agar and incubated at 30 °C overnight. Initial identification of isolated bacteria was performed using colony polymerase chain reaction (PCR) with the primers 27f and 1525r (Chanbusarakum and Ullman 2008). To identify BFo bacteria, sequenced amplicons were used, along with 16S sequences published previously for BFo bacteria (Chanbusarakum and Ullman 2008) to reconstruct a Neighbor-Joining (NJ) phylogenetic tree. Positive identification of BFo bacteria was assumed for those colonies that generated 16S amplicons that clustered together in the tree with previously published sequences from BFo bacteria. All strains isolated by ourselves and those used for genomic comparisons throughout this study are listed in table 1.

**Table 1:** Genomic Features of BFo Bacteria and Closely Related Species

Species	Estimated Genome Size (Mb)	Average Whole-Genome GC Content (% mol)	No. Genes	Predicted Pseudogenes	Total RNAs	Plasmids	Status	Source	GenBank Accession	Plasmid(s) Accession
BFo										
BFo1 (Netherlands)	5.13	51.82	4,829	98	76	—	Draft	This study	JMS00000000	—
BFo1 SwAb130 (Netherlands)	5.17	54.59	4,914	264	75	—	Draft	This study	LAGP00000000	—
BFo1 Keele2 (UK)	5.01	54.41	4,670	252	63	—	Draft	This study	LAGQ00000000	—
BFo2 Swan1 (Netherlands)	3.10	45.72	3,068	101	53	—	Draft	This study	JMSP00000000	—
BFo2 Swan69 (Netherlands)	3.24	46.34	3,136	185	61	—	Draft	This study	LAGS00000000	—
BFo2 Keele1 (UK)	2.70	46.9	2,681	128	45	—	Draft	This study	LAGR00000000	—
*Erwinia*										
*E. amylovora* ATCC49946	3.81	53.6	3,583	46	100	2	Complete	Sebaihia et al. (2010)	NC_013971	NC_013972.1; NC_13973.1
*E. amylovora* CFBP1430	3.83	53.6	3,455	21	100	1	Complete	Smits et al. (2010b)	NC_013961	NC_013957.1
*E. amylovora* LA637	3.87	53.7	3,565	2	58	2	Draft	Unpublished	NZ_CBVU000000000	NC_023056.1; NC_023072.1
*E. amylovora* LA636	3.79	53.6	3,471	2	58	—	Draft	Unpublished	NZ_CBVT000000000	—
*E. amylovora* ACW56400	3.86	53.5	3,909	13	66	1	Draft	Unpublished	NZ_AFHN00000000	NC_018999.1
*E. pyrifoliae* Ep1/96	4.07	53.4	3,728	50	97	4	Complete	Kube et al. (2010)	NC_012214	NC_013264.1/FP236827.1; NC_013265.1/FP236828.1; NC_013954.1/FP928999.1; NC_013263.1/FP236829.1
*E. pyrifoliae* DSM 12163	4.07	53.4	3,725	75	97	4	Complete	Smits et al. (2010a)	NC_017390	NC_017391.1/FN392236.1; NC_017388.1/FN392237.2; NC_017392.1/FN392238.1; NC_017389.1/FN392239.1
*E. tasmaniensis* Et1/99	4.07	53.4	3,721	57	103	5	Complete	Kube et al. (2010)	NC_010694	NC_010695.1; NC_010696.1; NC_10697.1; NC_010699.1; NC_010693.1
*E. toletana* DAPP-PG 735	5.27	53.6	4,812	48	50	—	Draft	Passos da Silva et al. (2013)	NZ_AOCZ00000000	—
*E. billingiae* Eb661	5.37	55	4,928	39	98	2	Complete	Kube et al. (2010)	NC_014306	NC_014304.1; NC_014305.1
*E. billingiae* Ep1/96	4.00	53.4	3,645	57	82	4	Complete	Kube et al. (2010)	FP_236843	FP_928999; FP236827; FP236828; FP236829
*E. tracheiphila* PSU-1	4.72	50.1	4,314	487	54	—	Draft	Unpublished	NZ_APJK00000000	—
*Pantoea*										
*Pantoea dispersa* EGD-AAK13	4.77	57.8	4,382	15	71	—	Draft	Unpublished	NZ_AVSS00000000	—
*P.* sp. At-9b	6.31	54.3	6,008	—	107	5	Complete	Unpublished	NC_014837	NC_014838.1–NC_01482.1
*P. agglomerans* IG1	4.83	55	4,443	32	65	—	Draft	Matsuzawa et al. (2012)	NZ_BAEF00000000	—
*P. agglomerans* 299R	4.58	54.3	4,320	41	90	—	Draft	Remus-Emsermann et al. (2013)	NZ_ANKX00000000	—
*P. agglomerans* Tx10	4.86	55.1	4,482	17	95	—	Draft	Smith et al. (2013a)	NZ_ASJI00000000	—
*P. agglomerans* Eh318	5.04	54.8	4,791	55	101	—	Draft	Unpublished	NZ_AXOF00000000	—
*P. agglomerans* strain 190	5.00	55.1	4,621	58	101	—	Draft	Lim et al. (2014)	JNGC00000000	—
*P. agglomerans* RIT273	5.37	55.1	4,927	32	93	—	Draft	Unpublished	NZ_JFOK00000000	—
*P. agglomerans* DAPP-PG734	5.37	54.7	5,107	57	106	—	Draft	Moretti et al. (2014)	NZ_JNVA00000000	—
*P. agglomerans* strain 4	4.81	55.1	4,370	37	70	—	Draft	Unpublished	JPOT00000000	—
*P. vagans* C9-1	4.89	55.1	4,512	101	99	2	Complete	Smits et al. (2010c)	NC_014562	NC_014561.1/CP001893.1; NC_014563.1/CP001894.1
*P. vagans* MP7	4.59	55.3	4,125	—	—	—	Draft	Unpublished	N/A	—
*P. ananatis* LMG 5342	4.91	53.3	4,525	70	99	1	Draft	De Maayer et al. (2012)	NC_016816	NC_016817.1/HE617161.1
*P. ananatis* LMG2665	4.98	53.4	4,624	47	88	—	Draft	Adam et al. (2014)	NZ_JFZU00000000	—
*P. ananatis* AJ13355	4.88	53.7	4,138			1	Complete	Hara et al. (2012)	NC_017531.1	NC_017533.1/AP012033.1
*P. ananatis* BI-9	5.11	53.5	4,759	32	75	—	Draft	Kim et al. (2012)	NZ_CAEI00000000	—
*P. ananatis* DAR76143	5.25	53.4	4,914	792	64	—	Draft	Unpublished	NZ_BATH00000000	—
*P. ananatis* Sd-1	4.92	53.3	4,548	72	73	—	Draft	Unpublished	GCF_000582575.1	—
*P. ananatis* BRT175	4.85	53,.7	4,696	38	95	—	Draft	Smith et al. (2013b)	NZ_ASJH00000000	—
*Tatumella*										
*Tatumella ptyseos* ATCC 33301	3.53	51.6	3,349	—	—	—	Draft	Tracz et al. (2015)	ATMJ00000000.1	—
Other										
*Plautia stali* symbiont	3.77	57	4,062	1,202	75	2	Complete	Kobayashi et al. (2011)	NC_022546	NC_022533.1; NC_022534.1

Note.—Genomic features of BFo bacteria and a comparison with representative individuals from closely related species. Table includes species from the genus *Erwinia*, *Pantoea,* and *Tatumella* and also includes the closely related symbiont from *Plautia stali.* Table shows genome size (Mb), number of plasmids (along with GenBank accession number), average whole-genome G+C content, number of genes, pseudogenes, and RNAs. Total RNAs do not include noncoding RNAs. An indication of the sequencing status of the genome is also given. For BFo bacteria, origin of *F. occidentalis* population, where isolated, is given in parenthesis along with GenBank accession numbers. “—” indicates value unknown.

### De Novo Genome Sequencing

Bacteria isolated from *F. occidentalis* were grown in LB until mid-log phase at 30 °C with shaking (250 rpm). Genomic DNA was extracted from liquid cultures using the QIAmp mini kit (Qiagen). Genomic DNA libraries were prepared for sequencing using Illumina Nextera XT Sample Preparation technology. Libraries were sequenced using a combination of MiSeq runs utilizing V2 and V3 MiSeq reagent kits for 300, 500 (V2), and 600 (V3) cycle runs. The paired-end sequencing reads (approximately 1,650,000; BFo1 and 1,270,000; BFo2) were preprocessed using the FASTX-trimmer in the FASTX-toolkit (http://hannonlab.cshl.edu/fastx_toolkit/, last accessed July 8, 2015). Reads were trimmed such that 20 bases were removed from the 3′-ends. Low-quality base calls were removed prior to assembly of the reads into contigs using the Velvet genome assembler (Zerbino and Birney 2008). To check for sequencing errors and quality, isolates taken from the Netherlands population were resequenced independently at Aberystwyth University as follows: Nextera XT Libraries were prepared from 1 ng of genomic DNA. Libraries were sequenced on a MiSeq (Illumina) using 2 × 151 bp reads; generating 1,031,616 (BFo1) and 419,060 (BFo2) paired-end reads. De novo assembly of reads was performed in CLC Genomics Workbench version 6.5.1. Genome metrics for all isolates are shown in table 1. Genome sequencing was performed in duplicate on two separate isolates per symbiont, per population. Assembly and subsequent filtering for contamination with the PGAP pipeline resulted in the generation of the following contigs: BFo1 SwAb130 (130), Keele2 (191), Netherlands (528) and BFo2 Swan69 (68), Keele1 (201), Netherlands (Swan1) (757).

Contigs generated for each BFo isolate were ordered using Mauve Contig mover (Rissman et al. 2009). For BFo1, contig order was determined using *E. pyrifoliae* and *E. tasmaniensis* as reference genomes. Due to the fact that phylogenetic placement of BFo2 has been a source of conflict and our phylogeny was unable to identify a closely related species to use as a reference genome, contigs for this species were not reordered. Annotation of all draft genomes was undertaken using both RAST and the PGAP pipeline (Tatusova et al. 2013). Similarity within strains of BFo1 and BFo2 was calculated using in silico DNA–DNA hybridization (DDH) implemented with the Genome-to-Genome Distance Calculator (Auch et al. 2010).

### Phylotyping of BFo Isolates

Closest relatives to BFo isolates were also identified with RAST (Aziz et al. 2008) using a comparison of 8–15 genes, by phylotyping using 31 protein-encoding genes in AMPHORANET (Wu and Eisen 2008) and by submitting the whole-genome sequences to the online ribosomal multilocus sequence typing (rMLST) database (Jolley et al. 2012). In the latter approach, 53 conserved genes encoding bacterial ribosome protein subunits (*rps* genes) are identified and compared with all bacterial sequences in the database. As of March 2015, the rMLST database contains approximately 113,000 whole bacterial genomes.

### Phylogenetic Classification of BFo Bacteria

Previous studies that have relied solely on rRNA genes to classify species of *Erwinia* and *Pantoea* have had mixed success and it has been shown that 16S rRNA phylogenies of the genus *Pantoea* can be of poor resolution (Rezzonico et al. 2009). Thus, we took advantage of our whole-genome sequences of BFo1 and BFo2 to undertake a multifaceted classification. To improve on existing, single-gene classifications of BFo1 and BFo2 phylogenetic reconstructions were performed as follows. First, to confirm phylogenetic placement of both BFo isolates within the enterobacteriales, phylogenies were reconstructed using multilocus sequence analysis (MLSA). First, a phylogeny using a concatenation of five slowly evolving protein sequences (Frr, NusA, PgK, RpmA, and RpsS) was reconstructed. Concatenation was performed on BLASTP (Altschul et al. 1990) retrieved orthologs from 74 taxa within the enterobacteriales using protein sequences from BFo bacteria as queries. Individual protein sequences were aligned in MEGA (Molecular Evolutionary Genetic Analysis v.6; Tamura et al. 2013) and all alignments concatenated into a supermatrix using the Perl script FASCONCAT (Kuck and Meusemann 2010). The final datamatrix contained 74 taxa and an alignment of 1,379 amino acids. An amino acid phylogeny was chosen to circumvent the problems often associated with saturation of the third codon position in nucleotide phylogenies with deep branches. In addition, the five proteins were chosen because of their availability in many whole and partially sequenced enterobacterial genomes. To ensure gap-free alignments, species were removed if no orthologs for one of the proteins could be retrieved from its genome—however, when this occurred another representative from that genus was still present in the data set. Second, a four-gene MLSA using the housekeeping genes *gyrB, rpoB, infB,* and *atpD* was conducted from members of the *Pantoea*, *Erwinia,* and *Tatumella* genera (112 taxa; 2,620 nt). Gene sequences were chosen because of their previous use at elucidating relatedness among species in the Enterobacteriaceae (Brady et al. 2013). In both phylogenies, sequence alignments were performed using ClustalW, implemented in MEGA (Molecular Evolutionary Genetic Analysis v.6; Tamura et al. 2013) Concatenation of the individual alignments was performed using the Perl script FASCONCAT (Kuck and Meusemann 2010). Phylogenies were reconstructed using two algorithms—first a maximum-likelihood (ML) protein phylogeny was constructed in MEGA for the five-gene MLSA using the LG model (Le and Gascuel 2008) with a gamma distribution of five discrete gamma categories a +G parameter of 0.695 (LG + G). The branch leading to *Dickeya* was used to outgroup root the phylogeny. Reconstruction of the four-gene MLSA phylogeny was performed in MEGA using the general time reversible (GTR) model with a gamma distribution and proportion of sites invariable (G+I). *Pectobacterium* sp. (gamma-Proteobacteria) was used to outgroup root the phylogeny. In all cases, bootstrap analysis (1,000 pseudoreplicates) was used to infer nodal support. Second, a Bayesian Inference (BI) phylogeny was also reconstructed for each MLSA data set. Bayesian phylogenies were reconstructed using MrBayes (Ronquist et al. 2012) run on the Cipres Science Gateway Xsede server (Miller et al. 2015) using the following parameters: Protein model or nucmodel= 4by4, rates= equal, Nst=1, Nbetacat= 5, and default priors.

Finally, whole-genome-based phylogenies were constructed as a result of a reference pan-genome approach (Meric et al. 2014). Briefly, a preliminary BFo reference pan-genome was created containing “unique” loci from all strains of BFo1 and BFo2. To do so, automatic annotations of each individual BFo1 and BFo2 assembled genomes were obtained using RAST. To empirically determine the optimum percentage coding length to retrieve allelic variants without spurious hits, we used the following parameters: 70% nucleotide sequence identity over 1) 10%, 2) 50%, and 3) 70% of the coding sequence (CDS) length. Despite using three different parameters our core genomes differed by only nine genes. Thus, we opted for the more relaxed criteria with allelic variants being defined as genes with more than 70% nucleotide sequence identity on more than 10% of the coding length. Hits were subsequently filtered out using Basic Local Alignment Search Tool (BLAST) (Meric et al. 2014), thus creating a list of 7,090 genes. The prevalence of these genes was examined in the genomes of all BFo isolates, and to an initial list of 50 *Erwinia*, *Dickeya,* and *Pantoea* genomes using the tools implemented in BIGSdb (Jolley and Maiden 2010). Species of *Dickeya* were included in the analysis as these are reports of plant phytopathogens in this genus (Khayi et al. 2014) and an earlier reclassification of *Erwinia chrysanthemi* to *Dickeya dadantii*. A total of 1,040 homologous genes were identified between BFo1 and BFo2. Concatenated gene-by-gene alignments of these (Sheppard et al. 2012) were produced using MAFFT (Katoh et al. 2002) and used to create phylogenetic trees using the NJ algorithm implemented in FastTree (Price et al. 2010).

### Construction of the *Pantoea/Erwinia* Last Common Ancestor Core Genome

To reconstruct the most probable evolutionary history of the BFo genomes after the split from *Pantoea/Erwinia*, core genomes for the *Pantoea* and *Erwinia* genera were reconstructed and compared with the genome of BFo2. Briefly, core genomes for five completely sequenced *Erwinia* genomes (*E. amylovora, E. tasmaniensis, E. pyrifoliae, E. billingiae,* and *E.* sp strain EJp617) and three completely sequenced *Pantoea* (*P. ananatis, P. vagans,* and *P.* sp. At-9b) were reconstructed independently using an all-against-all reciprocal BLAST approach. Briefly, each CDS was used as a BLAST query against a local database of CDS from all species outlined above. Presence of true orthologs of a particular gene was recorded if during pairwise reciprocal BLAST that gene returned as the best hit in both species. To avoid complications of gene duplication, we restricted the analysis to a presence-and-absence of orthologs approach—thus, in the case of gene duplication, the number of paralogous copies was not taken into account during the analysis. The putative ancestral core genome for the Last Common Ancestor (LCA) of *Erwinia* and *Pantoea* was reconstructed by retaining those genes that were conserved in these genus-specific core genomes. Comparisons were made between BFo genomes and the ancestral core genome.

### Nucleotide Substitution Rates

Data sets of orthologous gene pairs between BFo strains and *E. tasmaniensis* and *E. pyrifoliae* were constructed using a reciprocal BLAST approach. Pairwise alignments were performed in MEGACC (Kumar et al. 2012) with low-quality, gap-rich alignments removed manually. Pairwise estimates of the synonymous (d*S*) and nonsynonymous (d*N*) substitution rates were obtained for all retained gene pairs using the program YN00 (PAML; Yang 1997). Plots of d*N*/d*S* were generated to compare intact orthologs among BFo and closely related species.

## Results

### Classification of BFo1 and BFo2 Using a Multifaceted Approach

**Fig. 1.—:**
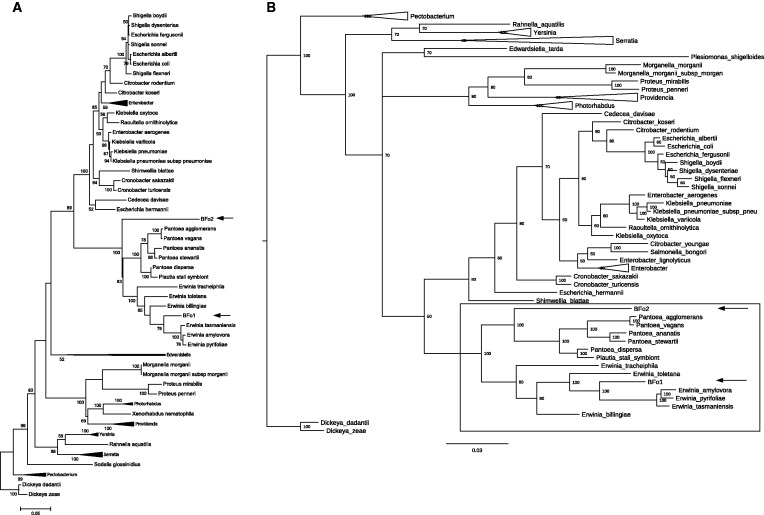
Phylogenetic reconstruction showing placement of BFo bacteria among closely related Enterobacteriales. Phylogenetic tree constructed using a 5-protein MLSA. Gap-free alignment of five protein sequences giving a total of 833 positions. (*A*) ML consensus tree was constructed using the LG+G model with five categories and a +G parameter of 0.695. The initial tree for heuristic searching was obtained using the NJ algorithm. Bootstrap analysis with 1,000 pseudoreplicates was performed to infer nodal support. Bootstrap values below 70% not shown. Homogenous clades have been collapsed. Arrows indicate BFo. (*B*) 50% majority rule consensus BI phylogeny using the model=protein, Nst=1, Nbetecat=5, and default priors. Numbers at nodes indicate percentage node probably. Probabilities <50% not shown. Shaded area indicates the positions of the *Pantoea* and *Erwinia* genera (+both BFo bacteria). Homogenous clades have been collapsed. Arrows indicate BFo. Both trees are outgroup rooted along the branch leading to *Dickeya*.

**Fig. 2.—:**
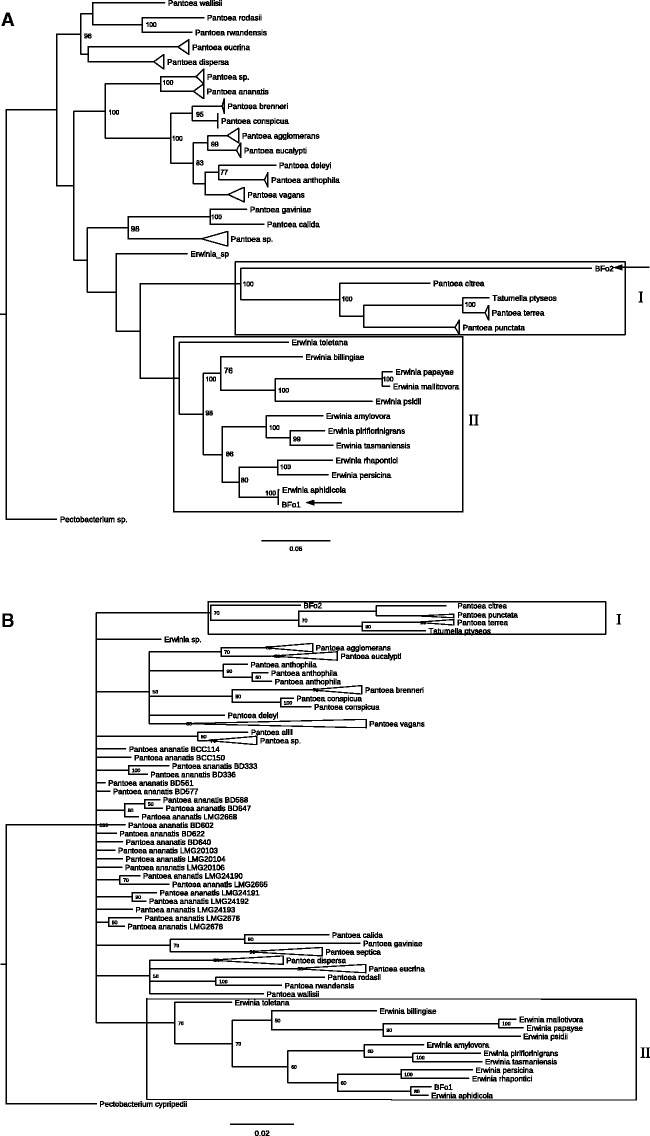
Phylogenetic reconstruction using a four-gene MLSA of BFo bacteria and closely species of *Pantoea* and *Erwinia*. Phylogenetic reconstruction using a four-gene MLSA. Gap-free alignment of four housekeeping gene sequences giving a total of 2,620 nt positions. (*A*) ML tree was constructed using the GTR model with five categories and a +G parameter of 0.695. The initial tree for heuristic searching was obtained using the NJ algorithm. Bootstrap analysis with 1,000 pseudoreplicates was performed to infer nodal support. BFo2 species indicated by arrows. Japanese *Pantoea* (+Tatumella) indicated by box I. *Erwinia* indicated by box II. Branches with greater than two identical strains/species have been collapsed. Bootstrap values below 75 not shown. (*B*) 50% majority rule consensus BI phylogeny using the nucmodel 4by4, Nst=1, Nbetecat=5, and default priors. Numbers at nodes indicate percentage node probably (only probabilities >50% shown). Japanese *Pantoea* (+*Tatumella*) and *Erwinia* demarcated with boxes (I and II, respectively). Branches with greater than two identical strains/species are collapsed.

Consistent with previous studies, plating of the homogenate obtained from surface sterilized *F. occidentalis* in all populations revealed two predominant colony morphologies. A very small number of non-BFo colonies were also isolated, typing these by 16S PCR indicated that they were related to the endosymbiont of *Nilaparvata lugens* and bacteria isolated from the gut of *Apis cerana japonica* (GenBank accession numbers of 16S sequence: JQ975877 and AB668063, respectively). However, growth of these was not consistent between isolations and thus was not addressed in depth. Identification of BFo-like colonies was performed using a combinatorial approach of 16S colony PCR and reconstruction of an NJ phylogeny (supplementary fig. S1, Supplementary Material online). This showed that our isolates cluster together with previously identified BFo bacteria. All data analysis was performed on several strains of BFo1 and BFo2 (table 1); however, they all appeared to group together in our phylogenies, thus will be referred to collectively as BFo1 and BFo2 hereafter. Moreover, in silico DDH indicated that the probability that all BFo1 isolates being the same species and all BFo2 isolates being the same species (i.e., DDH > 70%) was always greater than 97%. Additional classification, using ML and BI phylogenies reconstructed from 833 aligned and concatenated amino acid positions from 83 species (fig. 1*A* and *B*, respectively) and ML and BI reconstructed phylogenies of four gene sequences (2,620 nt positions, 115 species; fig. 2*A* and *B*, respectively), shows that BFo1 clusters within the genus *Erwinia.* Evidence from both trees suggests a common ancestor with the phytopathogens *E. amylovora*, *E. pyrilifoliae,* and the nonpathogenic *E. tasmaniensis*. More specifically, the four-gene MLSA tree indicates that BFo1 may be closely related to *E. aphidicola*. In contrast, both data sets also show that BFo2 is a member of the Enterobacteriacae but both data sets also position BFo2 on a separate branch adjacent to the *Pantoea* clade. Interestingly, both data sets also position BFo2 as a sister branch to the “Japanese” *Pantoea* (rather than the “core” *Pantoea*). BFo species are also found in distinct sequence clusters from other phytopathogens and several other endosymbionts. The deep branching of BFo2 from *Pantoea/Erwinia* sensu stricto is consistent with a prolonged period of independent evolution.

Despite being more polytomous, trees reconstructed using BI showed similar topologies to the ML reconstructions but with very minor rearrangements.

**Fig. 3.—:**
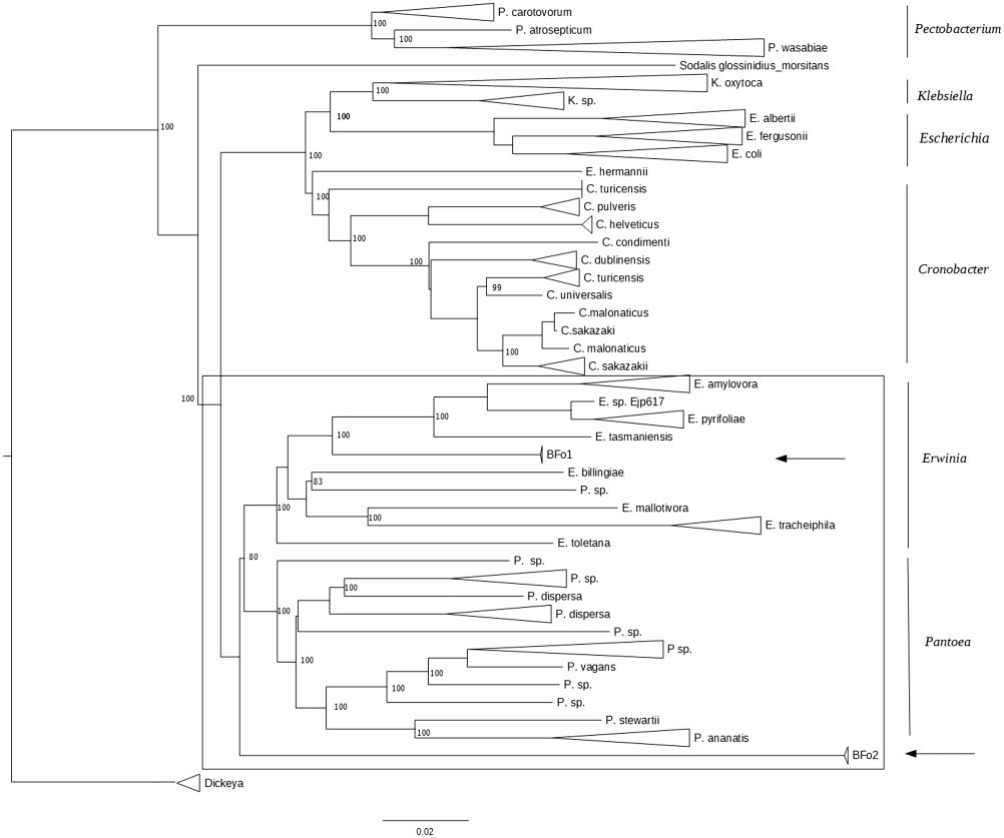
Core genome phylogeny of BFo bacteria and closely related Enterobacteriales. NJ phylogenetic tree reconstructed using gene-by-gene alignments from genes shared by 90% of 141 whole bacterial genomes. Position of BFo1 and BFo2 strains are indicated with an arrow. Tree is rooted along the branch leading to *Dickeya*. Scale bar indicates genetic distance. Bootstrap values indicate nodal support from 1,000 pseudoreplicates. Boxed area shows the *Erwinia/Pantoea* sensu stricta.

To support our initial findings, all BFo isolates were phylotyped to identify closely related species using online annotation and classification severs. Both AMPHORANET and RAST returned *E. tasmaniensis* as the most closely related species to BFo1 and *Pantoea* sp. At-9b as the most closely related species to BFo2. Additionally, a whole-genome NJ phylogeny of concatenated gene alignments from loci shared by 90% of 141 enterobacterial genomes, respectively, was also conducted (fig. 3). The topology of the resultant tree was consistent with that of both MLSA trees with BFo1 positioned within the *Erwinia* clade. Again, BFo2 was positioned at the end of a long branch outside of the *Erwinia* and *Pantoea* genera. Perhaps, the biggest difference was the positioning of BFo2 as an outgroup to the *Erwinia/Pantoea* genera in the five-gene MLSA and core genome phylogenies (figs. 1*A*, *B*, and 3) and the grouping of BFo2 more closely with species of *Pantoea* (four-gene MLSA; fig. 2*A* and *B*). Discrepancies between these data sets are likely the result of differing phylogenetic signal among the markers. rMLST analysis clustered BFo1 with *Erwinia*. In this case, all *rps* genes retrieved from the genomes of BFo1 returned best hit alignments to orthologous *rps* genes in *E. pyrifoliae*. In contrast, only a single *rps* gene produced any significant alignment (from *P. ananatis*; 100% query coverage; 90% identity) to orthologous *rps* genes in BFo2. None of the other *rps* genes in the BFo2 genome produced any significant hits, despite the database containing 105,000 whole bacterial genomes.

### Genome Comparisons of BFo Bacteria and Closely Related Species

The Whole-Genome Shotgun projects of BFo1-like and BFo2-like isolates described in this article have been deposited at DDBJ/EMBL/GenBank as individual Biosamples under the multispecies BioProject PRJNA23451 (table 1). The average genome size for BFo1 and BFo2 strains was 5.1 and 3.01 Mb, respectively. Within species genome sizes were consistent except for BFo2 isolated from the Keele (UK) population of *F. occidentalis*—which was slightly smaller. A comparison of genome sizes between BFo bacteria and closely related bacteria (table 1) shows that the genomes of the BFo1 strains in this study are consistent with those of many nonphytopathogenic *Erwinia.* More specifically, BFo1 shares similar genome sizes to *E. amylovora* and *E. tasmaniensis*. Conversely, average genome sizes for BFo2 strains are slightly smaller than those of the *Erwinia* and *Pantoea* genera.

### Secretion Systems and Virulence Factors

Searches for virulence factors and pathogenicity determinants that are ubiquitous in *Erwinia* were performed. These included three secretion systems (T2SS, T3SS, and T6SS), the exopolysaccharide gene clusters of amylovoran (*ams*), stewartan (*cps*) and levansucrase (lscC), the sorbitol and sucrose operons, and two flagella biosynthesis loci. BFo1 isolate genomes in this study lacked both the ancestral Hrp-T3SS and Inv/Spa-type T3SS—evidenced by the absence of orthologs of *hrpANW, dspE/A* (Hrp) and the Inv–Spa type secretion systems found in PAI-2 and -3 of *E. amylovora*. However, the genomes of BFo1 are predicted to encode three previously described T6SS loci (T6SS-1, T6SS-2, and TSS-3). The three T6SS loci in BFo1 also show a high degree of gene order conservation when compared with other species of *Erwinia*. Additionally, the genome of BFo1 contains the exopolysaccharide gene cluster for amylovoran of which they share greatest amino acid similarity (>80%) to the glycoside transferases found in *E. tasmaniensis.* Gene clusters for levansucrase and stewartan were absent—despite their presence in *E. amylovora* and *E. pyrifoliae*. Moreover, the genome of BFo1 also encodes two flagella biosynthesis loci (*flg-1* and *flg-2*). In comparison, BFo2 lacks the T6SS secretion systems, which is evidenced by the absence of orthologs for genes predicted to encode ImpDEFILM as well as the lipoprotein VasD. Moreover, the genome of BFo2 (unlike BFo1) lacks orthologs of *impK* (*dotU*) and *vgrG.* Similarly, BFo2 also lacks an ortholog of *outO,* which is a core component of the T2SS. The genomes of BFo2 strains also do not encode any of the exopolysaccharides commonly secreted by *Erwinia* and *Pantoea* species.

### Comparison of the Putative LCA Core Genome and the BFo2 Genome

Our reconstructed core genomes highlighted 2,080 and 3,464 conserved genes in the *Erwinia* and *Pantoea* isolates in this study, respectively. Of these two core genomes, we uncovered 1,967 genes that were conserved among these two genera—thus representing a core putative genome of the *Pantoea/Erwinia* LCA (supplementary table S1, Supplementary Material online). A comparison of the conserved genes in the putative LCA and the BFo2 genome revealed that BFo2 retains 1,753 of these, indicating that since the split from the LCA, BFo2 has lost at least 214 genes from its core genome (supplementary table S2, Supplementary Material online). Of these, 75 were annotated as hypothetical and 37 were related to metabolism and transport of amino acids, carbohydrates, and coenzymes. In addition, losses of genes were noted that are associated with signal transduction (four genes), DNA replication, recombination and repair (four genes), virulence (ten genes), motility (seven genes), posttranslational modification (eight genes), and cell wall/membrane biogenesis and cell division (eight genes). This is in contrast to BFo1; comparisons of this genome with the core genome of the LCA show that BFo1 has a deficit of only 13 genes conserved in the putative LCA core genome (supplementary table S3, Supplementary Material online).

### Molecular Evolution in BFo Bacteria

**Fig. 4.—:**
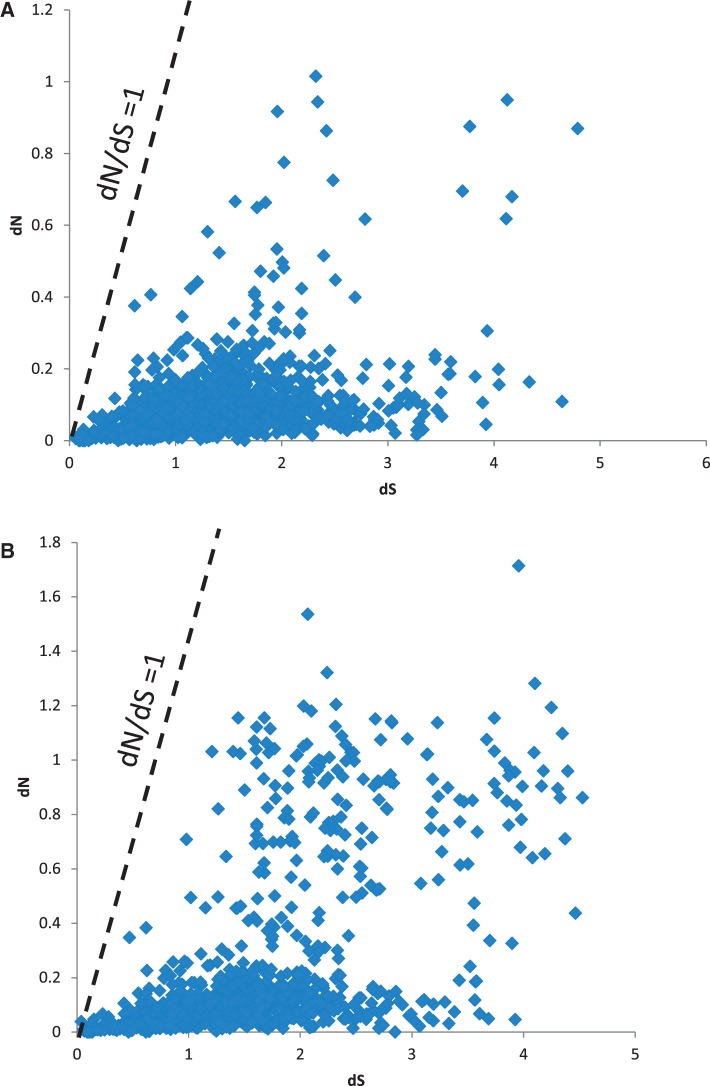
Plot of d*N* versus d*S* of orthologous genes between BFo1 and *E. tasmaniensis.* Plot of d*N* versus d*S* for all orthologous gene pairs between the nonpathogen, free living *E. tasmiensis* and BFo1. (*A*) BFo1 isolated from UK population of *F. occidentalis*. (*B*) BFo1 isolated from the Netherlands population of *F. occidentalis*. Each point represents an orthologous gene pair. Dotted line indicates neutrality (d*N* = d*S*).

Genome-wide rates of synonymous (d*S*) and nonsynonymous (d*N*) substitutions for all BFo species and strains were calculated using pairwise alignments of orthologous genes from the closest nonpathogenic species (*E. tasmaniensis* and *E. billingiae*) and the closest plant pathogen (*E. pyrifoliae*) (fig. 4). Comparisons between BFo1 strains and *E. tasmaniensis* revealed an average d*S* of 1.5 (Netherlands BFo1) and 1.3 (UK BFo1) with similar numbers of genes in both BFo1 strains approaching saturation for d*S* (>3.0). Very few genes in BFo1 had d*N*/d*S* ratios greater than 1 indicating that the majority are under purifying selection. Comparisons of pairwise orthologs between BFo1 strains and *E. pyrifoliae* and *E. billingiae* revealed almost complete saturation of d*S* (>3.0). Similarly comparisons of the rate of nucleotide evolution between BFo2 and members of the *Erwinia* and *Pantoea* genera resulted in saturation of the synonymous nucleotide substitution rates (d*S* > 5).

## Discussion

### Phylogenetic Reconstruction Clusters BFo1 with Species of *Erwinia*

Previous studies have classified both *F. occidentalis* bacterial symbionts as members of the diverse bacterial family Enterobacteriaceae (Chanbusarakum and Ullman 2008). Their single-gene phylogeny, using 16S rRNA sequences, showed that BFo1 clustered with species of *Erwinia*, whereas BFo2 clustered more closely with *E. coli*. Here, based on multisequence concatenated phylogenies, whole-genome phylogeny and comparisons of ribosomal genes (rMLST), we have reanalyzed the phylogenetic placement of these symbionts. A five-gene MLSA using the amino acid sequences of slow evolving genes positions both symbionts within the Enterobacteriales. More, parochially, a four-gene MLSA using housekeeping genes indicated that BFo1 may be closely related to *E. aphidicola*—an insect symbiont previous isolated from the Pea Aphid (*Acyrthosiphon pisum*). This is interesting as *E. aphidicola* has been shown to infect and reduce yield of *Phaseolus vulgaris* (Harada et al. 1997; Marin et al. 2011). More specifically, *E. aphidicola* has been implicated in causing internervial chlorosis, necrotic pits, and rough roots (Marin et al. 2011). Such preliminary findings suggest that *F. occidentalis* might be carrying a symbiont related to a phytopathogen capable of reducing crop yield. Indeed such findings may have large agroeconomical implications considering the now pan-global biogeography of *F. occidentalis*. However, we advise caution when interpreting phylogenies of minimal gene sets—especially as their reliability has been questioned when comparing whole-genome equivalents (Stephan et al. 2014). Indeed, despite the damage caused by feeding and transmission of tospoviruses, *F. occidentalis* appears not to be implicated in the damage reported by Marin et al. (2011). Additionally, comparisons of biochemical properties between *E. aphidicola* and BFo1 conducted by Harada et al. (1997) and de Vries, Breeuwer, et al. (2001), respectively, indicate that *E. aphidicola* is able to utilize inositol, sorbitol and citrate, whereas BFo1 is not. Thus although BFo1 is perhaps closely related to *E. aphidicola*, inference of such a relationship based on a four-gene phylogeny coupled with discrepancies in biochemical data suggests that BFo1 is not *E. aphidicola*. However, a definitive answer lies in the sequencing the *E. aphidicola* genome.

The topology of our core genome phylogeny is consistent with previous work on *F. occidentalis* symbionts (de Vries, Breeuwer, et al. 2001). BFo1 clusters with plant phytopathogens in the genus *Erwinia* and is likely to have shared a common ancestor with *E. pyrifoliae/E. amylovora* and the nonpathogenic species *E. tasmaniensis*. Indeed, *E. amylovora* infects the leaves and flowers of plants in the Rosaceae (Vrancken et al. 2013) and these plants are common hosts of *F. occidentalis* (Rahman et al. 2010, 2011). The question of whether *F. occidentalis* continually acquires BFo1 from the environment is difficult to answer—however, the fact that both BFo bacteria have been isolated from populations of *F. occidentalis* across a wide geographical range and also from historical specimens (Chanbusarakum and Ullman 2008) suggests that they are both true symbionts and form a permanent association within the gut lumen of *F. occidentalis*.

### BFo1 Virulence Determinants and Common Ancestry with Nonpathogenic *E. billingiae* and *E. tasmaniensis*

A comparison of the virulence and pathogenicity factors encoded by three BFo1 genomes and closely related species of *Erwinia* gives some insight to the phylogenetic placements of these strains. A predicted virulence genotype, for an erwinial ancestor, has been characterized and changes in the gene repertoire in different lineages in relation to *Erwinia* radiation (Smits et al. 2011). In this study, the plesiomorphic genotype for this genus was predicted to contain an Hrp-like T3SS, a single flagellal locus (Flg-1), two T6SS loci (T6SS-1 and T6SS-2), two expolysaccharides (*lscC* and stewartan), and the sorbitol metabolism (*srl*) operon. The genome of BFo1 does possess three T6SS loci, of which T6SS-1 and T6SS-2 but does not possess other elements of the secretion system; possibly because it is more associated with infection of plants and causation of disease (Zhao and Qi 2011; Mann et al. 2013). Of those three T6SS loci, the first two are common in *Erwinia*, whereas the third is likely resultant of Horizontal Gene Transfer (De Maayer et al. 2011). Furthermore, neither of the two Inv/Spa-type T3SS, thought to have been present early on in the radiation of the genus, is present in BFo1. This pattern of gene gain or loss is consistent with frequent horizontal gene transfer associated with the radiation of *Erwinia* and *Pantoea* (De Maayer et al. 2011). Species of *Erwinia* also possess several metabolic factors that are considered to be important in causing disease in plants (Kube et al. 2010). These include exopolysaccharides—such as amylovoran, levan production, and the ability to synthesize sorbitol and sucrose. The BFo1 genomes sequenced in this study possess the amylovoran biosynthesis cluster (*ams*) but lack the ability to produce levan and do not contain the sorbitol or sucrose metabolism operons. A comparison of the *ams* gene cluster in BFo1 shows that it is most similar to that found in *E. tasmaniensis*. Principally, the *ams* gene cluster has been shown to be distinguishable from the Stewartan biosynthesis gene cluster in other species of *Erwinia* and *Pantoea* principally by the exchange of two glycoside transferases (annotated as *wbdN* and *cpsD*) at the center of the cluster (Kamber et al. 2014). Taken together, details of the predicted BFo1 virulence genotype, and estimated genome size, closely mirror that reported for *E. billingiae* Eb661 (Kube et al. 2010) and support evidence from genomic analysis that BFo1 may share a common ancestor with this species. However, this is not consistent with our core genome phylogeny that separates BFo1 from *E. billingiae.* The lack of an Hrp type III secretion system and T2SS within the BFo1 genome is consistent with a nonpathogenic lifestyle—as both the T2SS and T3SS are important features acquired during pathoadaption in *Erwinia* (Smits et al. 2013). Further characterization of the population structure of multiple BFo1 isolates will be necessary to confirm if the absence of an Inv/Spa-type T3SS is an ancestral trait, as in *E. pyrifoliae* and *E. tasmaniensis,* where this secretion system has been lost (Smits et al. 2013), and if comparable genome metrics with *E. toletana* can be instructive in understanding the evolution of this lineage.

### Phylogenetic and Genomic Analysis of BFo2

In contrast to the classification of BFo1, which clusters with the *Erwinia* genus, BFo2 isolated from the same host represents a relatively divergent lineage compared with the most closely related species based on protein and whole-genome phylogenies (figs. 1–3). Phylogenetic reconstruction with whole enterobacterial genomes indicates that it is somewhat divergent from known sequenced bacteria. Indeed, whole-genome alignments of BFo2 genomes and other enterobacteriales revealed very little genome conservation. Moreover, saturation of d*S* when comparing orthologous genes pairs in BFo2 and *Pantoea* and *Erwinia* provides some evidence of divergence of BFo2 from these two genera. The genome of BFo2 does not encode any of the commonly found secretion systems (T3SS and T6SS) in the *Pantoea* and *Erwinia* genera—the latter is evidenced by the lack of *impK* (*dotU*) and *vgrG* both of which are core components of the type VI secretion system (Broms et al. 2012). In addition BFo2 also appears also to lack genes of the T2SS—evidenced by the lack of an ortholog of *outO* which is a core component of the T2SS (Sandkvist 2001). The absence of these genes in BFo2 is perhaps consistent with gene loss and of ongoing evolution in an insect symbiont. The loss of secretion systems, including T2SS, T3SS and T6SS, can be beneficial to emerging symbionts—as their synthesis is energy demanding imposing a fitness cost and their absence could provide a selective advantage in environmental isolates (Gophna et al. 2003).

The BFo2 genomes also lacked 37 of the genes associated with transport and metabolism, 7 genes associated with motility, and 8 genes associated with cell wall/membrane biogenesis and cell division that we predicted in the *Erwinia/Pantoea* LCA core genome. The loss of so many genes involved in transport and metabolism of carbohydrates and amino acids in the BFo2 genome may indicate small-scale genome reduction as has been identified previously in a few insect symbionts such as *Ishikawaella* (Nikoh et al. (2011). Furthermore, it may suggest that BFo2 is reliant on BFo1 for full functionality. Consistent with this, BFo2 is rarely found in the gut of *F. occidentalis* without BFo1 (Chanbusarakum and Ullman 2009).

A comparative analysis of the genome metrics of BFo bacteria and members of the *Erwinia* and *Pantoea* genera reveal that although the genome size (in Mb) of BFo1 is consistent with closely related species, the genome of BFo2 is slightly smaller. A similar pattern is observed with %GC content, while BFo1 is consistent with the average *Erwinia* GC content, BFo2 is slightly lower. Such genome reduction and AT-bias in BFo2 may be indicative of an emerging facultative insect symbiont and is often associated with a long-term relationship with an eukaryotic host (Van Leuven and McCutcheon 2012). Indeed, although obligate insect symbionts, such as *Buchnera aphidicola,* with ancient relationships with their hosts often have reduced genomes, facultative symbionts, for example, *Candidatus Regiella insecticola* and *Candidatus Hamiltonella defensa* (Degnan et al. 2010), often have genomes that, while having undergone genome reduction, are much larger and have more dynamic genomes than endosymbiotic conspecifics (Engel and Moran 2013). Taken together, the reduced genome size, coding density, and reduced G+C content compared with closely related free-living species are consistent with BFo2 being a nascent symbiont*,* as observed in *Serratia symbiotica* (Burke and Moran 2011) and *Candidatus* Regiella insecticola in aphids (Degnan et al. 2010).

## Conclusions

Our analysis suggests that two of the primary bacterial symbionts isolated from *F. occidentalis* may represent novel species. Based on phylogenetic analysis, BFo1 could be described as an *Erwinia* species sharing a common ancestor with the nonpathogenic *E. tasmaniensis,* the epiphytic *E. billingiae,* and the pathogenic *E. pyrifoliae*. The grouping of BFo1 with *E. aphidicola* using a four-gene MLSA could be significant from an agroeconomical standpoint. But disparity between biochemical data among these two species and the lack of a WGS for *E. aphidicola* means that this relationship cannot be accurately elucidated as yet. Genome analysis of BFo1 reveals a lack of key genomic signatures that are commensurate with pathogenicity within this genus, consistent with a commensal life-style for BFo1. Phylogenetic classification of BFo2 revealed that these bacteria do not cluster closely with any available bacterial species genomes.

## Supplementary Material

Supplementary figure S1 and tables S1–S3 are available at *Genome Biology and Evolution* online (http://www.gbe.oxfordjournals.org/).

Supplementary Data
