# Supplementary material for: Draft Genomes, Phylogenetic Reconstruction, and Comparative Genomics of Two Novel Cohabiting Bacterial Symbionts Isolated from Frankliniella occidentalis
Source: Genome Biol Evol. 2015 Jul 21;7(8):2188–202. doi: 10.1093/gbe/evv136 (PMC4558854; doi:10.1093/gbe/evv136)
Supplement: Supplementary Data [file supp_7_8_2188__index.html]

Draft Genomes, Phylogenetic Reconstruction, and Comparative Genomics of Two Novel Cohabiting Bacterial Symbionts Isolated from Frankliniella occidentalis — Supplementary Data 

# Draft Genomes, Phylogenetic Reconstruction, and Comparative Genomics of Two Novel Cohabiting Bacterial Symbionts Isolated from *Frankliniella occidentalis*

## Supplementary Data

files

- Supplementary Data - docx file
- Supplementary Data - xlsx file
- Supplementary Data - pdf file
