# Supplementary material for: Draft Genomes, Phylogenetic Reconstruction, and Comparative Genomics of Two Novel Cohabiting Bacterial Symbionts Isolated from Frankliniella occidentalis
Source: Genome Biol Evol. 2015 Jul 21;7(8):2188–202. doi: 10.1093/gbe/evv136 (PMC4558854; doi:10.1093/gbe/evv136)
Supplement: Supplementary Data [file supp_evv136_New_Microsoft_Office_Word_Document.docx]

**Supplementary Table 1: Reconstructed *Erwinia/Pantoea* ancestral core genome**.

List of 1967 conserved genes predicted in the core genome of the putative *Erwinia/Pantoea* LCA. Accession numbers refer to orthologs in the *Erwinia billingiae* genome.

**Supplementary Table 2: Genes absent in the genome of BFo2 that were predicted in the core genome of the putative *Erwinia/Pantoea* LCA.**

Genes or hypothetical coding regions, grouped into COG functions that were present in the reconstructed core genome of the putative *Erwinia/Pantoea* LCA that were absent in BFo2. Accession numbers refer to orthologs in the *Erwinia billingiae* genome.

**Supplementary Table 3: Genes absent in the genome of BFo1 that were predicted in the core genome of the putative *Erwinia/Pantoea* LCA.**

Genes or hypothetical coding regions that were present in the reconstructed core genome of the putative *Erwinia/Pantoea* LCA that were absent in BFo1. Accession numbers refer to orthologs in the *Erwinia billingiae* genome.

**Supplementary Figure 1: NJ phylogeny of 16S sequences from bacteria isolated from Netherland and UK populations.**

Neighbor-Joining phylogeny of 16S sequences from bacteria isolated from two populations of F. occidentalis. Sequences from strains with prefixes TMV, TG1,TN1,TAC,TJA,TN2 and TWC were taken from (de Vries et al., 2008) those from Genbank are indicated in parentheses. Bacteria isolated during this study are positioned at the end of the two branches with labels “BFo*_x_* Netherlands and Keele”. Bootstrap values indicate nodal support from 1000 pseudoreplications.
