# Supplementary figures and images for: Draft Genomes, Phylogenetic Reconstruction, and Comparative Genomics of Two Novel Cohabiting Bacterial Symbionts Isolated from Frankliniella occidentalis
Source: Genome Biol Evol. 2015 Jul 21;7(8):2188–202. doi: 10.1093/gbe/evv136 (PMC4558854; doi:10.1093/gbe/evv136)

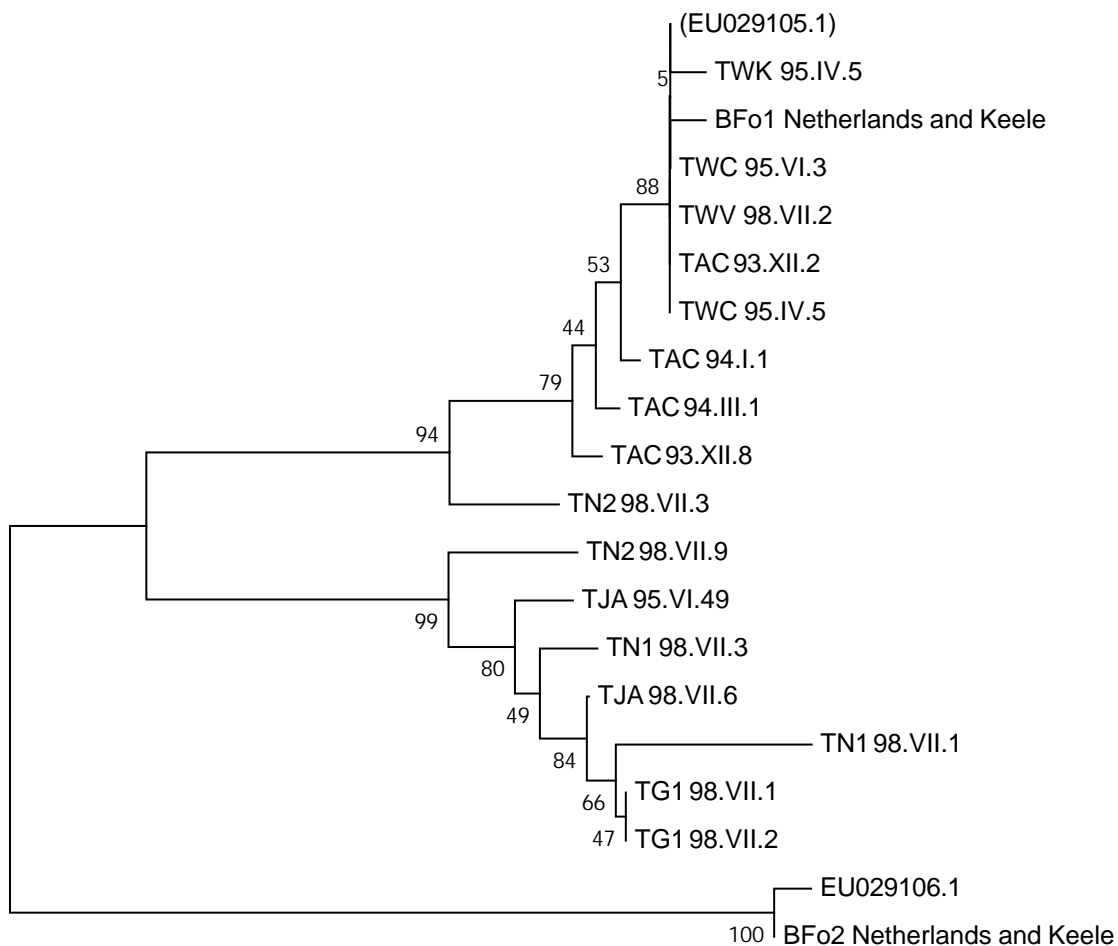

0.005

Supplement: Supplementary Data [file supp_evv136_Suppl_Figure_1.pdf]
